# Supplementary material for: Identification of photoperiod-induced specific miRNAs in the adrenal glands of Sunite sheep (Ovis aries)
Source: Front Vet Sci. 2022 Jul 22;9:888207. doi: 10.3389/fvets.2022.888207 (PMC9354845; doi:10.3389/fvets.2022.888207)
Supplement: Supplementary file 1 [file Data_Sheet_1.ZIP › Supplementary file/miRDeep2 file.zip!/miRDeep2 file/Know_Structure/SPLP42A2/oar-let-7d.pdf]

miRBase precursor : oar-let-7d
Total read count : 26711
oar-let-7d read count : 24704
remaining reads : 2007

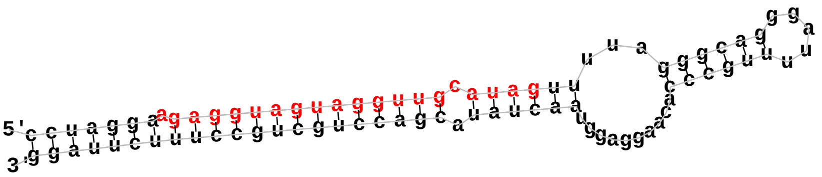

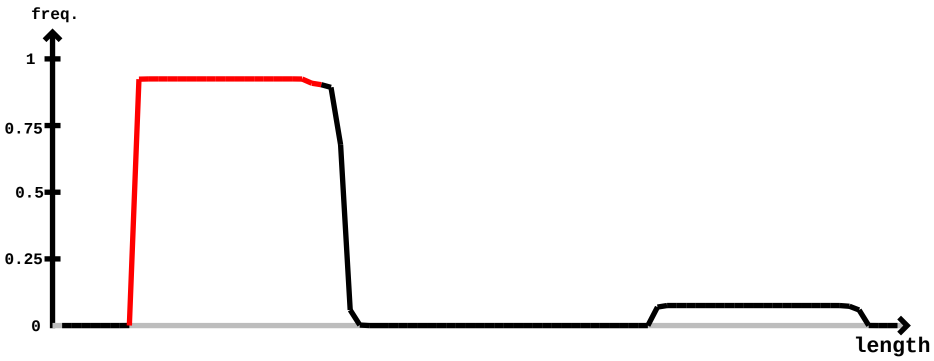


```
          oar-let-7d
5'- ccuaggaagagguaguagguugcauaguuuuagggcagggauuuugcccacaaggagguaacuauacgaccugcugccuuucuuagg   -3'    exp
    (((((((.((((((((((((((.((((((...((((((.....))))))..........)))))).)))))))))))))))))))))   reads      mm         sample
    ......aagagguaguagguugcauagu...........................................................   3          0          seq
    ......aagagguaguagguugcauaguu..........................................................   3          0          seq
    .......agagguaguagguugca...............................................................   12         0          seq
    .......agaggCaguagguugcau..............................................................   1          1          seq
    .......agagguaguagguugcau..............................................................   418        0          seq
    .......agagguaguagguugcaua.............................................................   147        0          seq
    .......agagguaguagguuAcaua.............................................................   1          1          seq
    .......agagguaguagguugcauag............................................................   265        0          seq
    .......agagguaguagguugcauagu...........................................................   5744       0          seq
    .......agagguaguagguugcauaguu..........................................................   16527      0          seq
    .......agagguaguagguugcauaguuu.........................................................   1505       0          seq
    .......agagguaguagguugcauaguuuu........................................................   53         0          seq
    ........gagguaguagguugcau..............................................................   1          0          seq
    ........gagguaguagguugcauagu...........................................................   3          0          seq
    ........gagguaguagguugcauaguu..........................................................   17         0          seq
    ........gagguaguagguugcauaguuu.........................................................   3          0          seq
    .........agguaguagguugcauaguu..........................................................   1          0          seq
    .............................uuagggcagggauuuugcccacaaggagguaa..........................   1          0          seq
    .............................................................cuauacgaccugcugcc.........   1          0          seq
    .............................................................cuauacgaccugcugccuu.......   1          0          seq
    .............................................................cuauacgaccugcugccuuu......   68         0          seq
    .............................................................cuauacgaccugcugccuuuc.....   347        0          seq
    .............................................................cuauacgaccugcugccuuucu....   1432       0          seq
    .............................................................cuauacgaccugcugccuuucuu...   3          0          seq
    ..............................................................uauacgaccugcugccuuu......   6          0          seq
    ..............................................................uauacgaccugcugccuuuc.....   22         0          seq
    ..............................................................uauacgaccugcugccuuucu....   122        0          seq
    ..............................................................uauacgaccugcugccuuucuu...   3          0          seq
    ...............................................................auacgaccugcugccuuucuua..   1          0          seq
```
